# Simplified Large-Scale Sanger Genome Sequencing for Influenza A/H3N2 Virus

**DOI:** 10.1371/journal.pone.0064785

**Published:** 2013-05-31

**Authors:** Hong Kai Lee, Julian Wei-Tze Tang, Debra Han-Lin Kong, Evelyn Siew-Chuan Koay

**Affiliations:** 1 Department of Pathology, Yong Loo Lin School of Medicine, National University of Singapore, Singapore; 2 Molecular Diagnosis Centre, Department of Laboratory Medicine, National University Hospital, National University Health System, Singapore; 3 Alberta Provincial Laboratory for Public Health, University of Alberta Hospital, Edmonton, Alberta, Canada; 4 Department of Medical Microbiology and Immunology, University of Alberta, Edmonton, Alberta, Canada; The Pirbright Institute, United Kingdom

## Abstract

**Background:**

The advent of next-generation sequencing technologies and the resultant lower costs of sequencing have enabled production of massive amounts of data, including the generation of full genome sequences of pathogens. However, the small genome size of the influenza virus arguably justifies the use of the more conventional Sanger sequencing technology which is still currently more readily available in most diagnostic laboratories.

**Results:**

We present a simplified Sanger-based genome sequencing method for sequencing the influenza A/H3N2 virus in a large-scale format. The entire genome sequencing was completed with 19 reverse transcription-polymerase chain reactions (RT-PCRs) and 39 sequencing reactions. This method was tested on 15 native clinical samples and 15 culture isolates, respectively, collected between 2009 and 2011. The 15 native clinical samples registered quantification cycle values ranging from 21.0 to 30.56, which were equivalent to 2.4×10^3^–1.4×10^6^ viral copies/µL of RNA extract. All the PCR-amplified products were sequenced directly without PCR product purification. Notably, high quality sequencing data up to 700 bp were generated for all the samples tested. The completed sequence covered 408,810 nucleotides in total, with 13,627 nucleotides per genome, attaining 100% coding completeness. Of all the bases produced, an average of 89.49% were Phred quality value 40 (QV40) bases (representing an accuracy of circa one miscall for every 10,000 bases) or higher, and an average of 93.46% were QV30 bases (one miscall every 1000 bases) or higher.

**Conclusions:**

This sequencing protocol has been shown to be cost-effective and less labor-intensive in obtaining full influenza genomes. The constant high quality of sequences generated imparts confidence in extending the application of this non-purified amplicon sequencing approach to other gene sequencing assays, with appropriate use of suitably designed primers.

## Introduction

In recent years, advances in sequencing techniques have enabled an increasing number of research studies based on the genome-wide sequences of the influenza viruses [1]–[6], rather than relying solely on an individual gene that may preclude more comprehensive gene signatures [7], [8]. Since the large number of influenza genome sequences deposited by Ghedin *et al*. [4] and the initiation of the Influenza Genome Sequencing Project in 2005 [9], the deposition of complete human influenza A virus genomes by other groups has increased exponentially.

The genome of the influenza A virus (family *Orthomyxoviridae*) consists of eight segmented, negative-stranded RNAs, ranging from 890 to 2,341 nucleotides (nt), constituting 13,627 nt per genome. The eight RNA segments encode for (in the order of the segment numbers one to eight): viral RNA polymerase basic 2 (PB2, 2341 nt), polymerase basic 1 (PB1, 2341 nt), polymerase acidic (PA, 2233 nt), hemagglutinin (HA, 1762 nt), nucleoprotein (NP, 1567 nt), neuraminidase (NA, 1466 nt), matrix (M1, 1027 nt), and nonstructural (NS1, 890 nt) protein. Apart from these proteins, alternatively spliced mRNAs of the seventh segment (M1) and the eighth segment (NS1) allow translation of two additional proteins, namely, the ion channel matrix protein (M2) and nuclear export/nonstructural protein (NEP/NS2), respectively. Also, PB1-F2 proteins are alternatively translated from PB1 gene segments of some influenza A viruses [10].

The introduction of next-generation sequencing (NGS), which delivers high throughput readings [11] compared to the traditional Sanger dideoxy chain-termination method [12], has provided a remarkable cost reduction for microbial genome sequencing. However, a higher error rate due to homopolymeric miscalling and other systematic base-calling biases have been observed in NGS techniques, compared with the Sanger methods [13]–[16]. The average error rate of the former is considerably higher, with a value of 10^−2^–10^−4^ versus that of the latter at 10^−4^–10^−5^ [13], [14]. A recent report on 12 influenza genomes comparing 2 NGS platforms from 454 Life Sciences and Illumina revealed error rates up to 10^−3^ and 10^−5^ at the homopolymeric region, respectively [17]. Besides, the cost of the initial NGS capital equipment outlay, together with the additional bioinformatics manpower support for the storage and analysis of the huge amount of data generated through the NGS system [18] may not be cost-effective for many smaller research laboratories for the sequencing of influenza viruses which have a relatively small genome size (∼14 kb).

The Sanger technique is regarded to be low throughput and more tedious, due to the requirement of multiple purification or plasmid cloning steps [4], [8], [19]–[23]. Here, we describe a whole genome sequencing method for seasonal influenza A/H3N2, with modifications of the normal sequencing protocol that reduces the number of processing steps, but still constantly produces a high quality sequence read of up to 700 bp. This protocol, when applied systematically, should hasten the routine genome sequencing work for local influenza surveillance studies. It was also demonstrated that this protocol is highly applicable for both clinical samples and Madin-Darby canine kidney- (MDCK-) cultured samples.

## Results

### Clinical Specimens and Culture Isolates

A total of 30 archived influenza A/H3N2 clinical samples collected from different patients between 2 May 2009-1 Aug 2011 were selected randomly for this study. All samples were received for diagnostic testing at the National University Hospital (NUH) in Singapore and were confirmed positive using two clinically validated, in-house, real-time influenza A/B screening [24] and subtyping assays [25], [26]. The samples included nasal/nasopharyngeal or throat swabs collected in universal transport medium, endotracheal tube aspirates, or sputum samples. Fifteen of the 30 were sequenced from cultured isolates of the original clinical sample using a MDCK.2 (ATCC; CRL-2936) cell line; the other 15 sequences were obtained directly from the clinical samples with no preliminary culture step.

### Primer Design

To ensure the utility of the assay for the sequencing of older as well as future circulating strains, two reference gene sequences were randomly chosen per month from depositions from different countries and dates of collection (2007 to 2011) available at the NCBI Influenza Virus Resource. Primer target regions for RT-PCRs for the different gene segments were selected from the conserved regions of the respective aligned gene sequences. Large gene segments (1 to 3) were amplified as three fragments. Small segments (4 to 8) were amplified as two fragments. To achieve tolerance for accurate sequence assembly, the PCR products for each of these segments overlapped with preceding and follow-up segments for at least 39 bp. The 5′ and 3′ ends of each segment were amplified using modified published forward (MBTuni-12) and reverse (MBTuni-13) primers [21], [27]. Sequencing primers were designed within the internal regions of the PCR products. All the sequencing and RT-PCR primers are listed in Tables 1 and 2, respectively.

**Table 1:** Summary of sequencing primers employed in this study and their respective performance.

Segment/fragment	Primers	Primer sequence (5′-3′)	Nucleotide position (5′-3′)	Reference	Average percentage of bases ≥QV40 (S.D.)	Average percentage of bases ≥QV30 (S.D.)	Mean LOR in bases (S.D.)
1(PB2)/A	PB2_230F25	CGGAGAGAAATGAACAAGGACAAAC	230–254	GU907121	91.62 (5.62)	94.46 (4.80)	556 (23)
	PB2_629R26	TCTCTCTAACATGTATGCAACCATCA	654–629		89.87 (7.45)	94.45 (5.05)	593 (24)
1(PB2)/B	PB2_960F21	CAARGCTGCAATGGGATTGAG	960–980		89.93 (5.82)	94.33 (4.69)	618 (23)
	PB2_1432R24	TCTCATTGACATCTCTGTGCTTGG	1455–1432		90.00 (6.36)	94.31 (4.83)	597 (24)
1(PB2)/C	PB2_1796F25	GCCAATACAGTGGGTTTGTCAGAAC	1796–1820		92.69 (3.74)	94.58 (3.37)	498 (17)
	PB2_2118R25	TCCRTAYCTTCTGTCTTCCTTACCT	2142–2118		89.27 (4.83)	93.79 (4.11)	580 (21)
2(PB1)/A	PB1_232F25	GATGGACCACTACCTGAGGATAATG	232–256	AB441948	91.70 (3.96)	94.56 (3.93)	540 (21)
	PB1_590R23	GGTCATGTTGTCYCTTACTCTCC	612–590		89.39 (5.70)	93.43 (4.77)	552 (24)
2(PB1)/B	PB1_1007F26	ATCAACCTGAGTGGTTCAGAAACATC	1007–1032		86.18 (5.45)	92.83 (4.38)	681 (23)
	PB1_1369R26	TCATGATYTGGTGCATTCACTATGAG	1394–1369		90.25 (5.11)	93.72 (4.52)	582 (26)
2(PB1)/C	PB1_1700F25	ATAGRTGCCATAGAGGAGACACACA	1700–1724		91.20 (3.87)	94.93 (3.49)	579 (22)
	PB1_2126R25	ATCGGTCTCCTATATGAACTACTAG	2150–2126		89.21 (6.23)	94.24 (5.56)	627 (31)
3(PA)/A	PA_210F24	GGTAGAACTTGACRATCCAAATGC	210–233	GU907117	90.40 (7.02)	93.96 (4.66)	520 (21)
	PA_601R23	GTTTCTTCGCCTCTTTCGGACTG	623–601		89.82 (5.04)	92.96 (4.66)	559 (25)
3(PA)/B	PA_862F23	TCCAARTTCCTCCTGATGGATGC	862–884		90.78 (3.85)	94.85 (2.96)	641 (18)
	PA_1225R24	CTGTAYCCAGCTTGAAAGTGACCT	1248–1225		91.55 (8.56)	94.21 (7.83)	493 (38)
3(PA)/C	PA_1608F20	TGACCCGAGAATTGAGCCAC	1608–1627		92.89 (3.16)	95.71 (2.73)	572 (13)
	PA_1975R24	AAATCCTTCCAATTGTGGTGATGC	1998–1975		90.37 (6.22)	93.16 (8.56)	544 (68)
4(HA)/A	HA_286F24	TATTGGGAGACCCTCADTGTGATG	286–309	GU907114	88.85 (5.64)	94.39 (3.70)	689 (16)
	HA_517R27	GGGTCAACCAATTCAATCTACTAAAGA	543–517		89.77 (6.92)	93.20 (6.23)	491 (22)
4(HA)/B	HA_1387F26	TTGATCTAACTGACTCAGAAATGAAC	1387–1412		88.61 (5.25)	91.77 (4.79)	324 (17)
	HA_1393R27	ACAGTTTGTTCATTTCTGARTCAGTTA	1419–1393		85.39 (14.55)	91.35 (10.33)	474 (18)
		ACAGTTTGTTCATTTCTGARTCAATTA	1419–1393				
	HA_1632R25	GCAAAAAACATGATATGGCAAAGGA	1656–1632		75.69 (11.79)	86.43 (8.36)	707 (26)
5(NP)/A	NP_166F25	ATCCAAATGTGCACTGAACTTAAAC	166–190	GU907120	88.28 (8.19)	94.00 (5.24)	653 (25)
	NP_664R20	CGYCCATTYTCACCTCTCCA	683–664		91.71 (5.63)	95.21 (4.06)	624 (23)
5(NP)/B	NP_998F25	CTAACGAGAATCCAGCACACAAGAG	998–1022		90.46 (4.60)	93.66 (4.17)	507 (20)
	NP_1322R23	CGTATTTCCAGTGAATGCTGCCA	1344–1322		88.65 (7.16)	93.33 (5.47)	520 (27)
6(NA)/A	NA_350F20	GGYGGRGACATCTGGGTGAC	350–369	GU907119	90.32 (4.46)	93.53 (3.39)	480 (17)
	NA_529R23	ATGCTATGCACACTTGCTTGGTC	551–529		88.24 (10.79)	92.38 (8.81)	494 (24)
	NA_699R25	CCATTGATACAAACGCATTCTGACT	723–699		87.70 (6.05)	93.66 (3.37)	667 (19)
6(NA)/B	NA_1090F24	AAATGACGTGTGGATGGGRAGAAC	1090–1113		88.12 (7.04)	91.74 (6.11)	322 (17)
	NA_1331R24	CACAACAATACTGTTYGAGGTCCA	1354–1331		90.43 (5.87)	94.36 (3.94)	584 (20)
7(MP)/A	MP_78F18	GCCCCCTCAAAGCCGAGA	78–95	GU907115	89.32 (5.56)	92.81 (1.93)	457 (17)
	MP_551R23	CTGGCCAARACCATTCTGTTCTC	573–551		90.42 (4.21)	94.10 (1.30)	520 (17)
7(MP)/B	MP_459F22	GYCTRGTATGTGCAACATGTGA	459–480		91.65 (2.20)	94.16 (1.75)	524 (11)
8(NS)/A	NS_38F23	CACTGTGTYARGTTTCCAGGTAG	38–60	GU907116	89.32 (6.65)	92.78 (4.77)	388 (16)
	NS_373R23	GATTGCCTGGTCCATTCTGATGC	395–373		89.32 (9.21)	92.40 (9.13)	340 (30)
8(NS)/B	NS462F24	TTACTAAGGGCTTTCACCGAAGAG	462–485		90.57 (5.73)	92.79 (5.48)	383 (21)
	NS795R25	AAACAGCAGTTGYAATGCTTGCATG	819–795		90.18 (2.32)	92.50 (2.31)	396 (9)

The performance of each sequencing primer is described in Table 1, as seen by the average percentage of bases generated from the 30 complete genomes with QV more than 30 and 40, respectively. The QV values were generated using the proprietary sequencing analysis software (version 5.2) of the ABI 3130×l genetic analyzer (Applied Biosystems). Length of Read (LOR) is defined as the length of sequence with QV20 and above for at least 20 continuous bases.

**Table 2:** PCR primers and second annealing temperatures (T_aS_) used to amplify the influenza A/H3N2 genome.

Segment/fragment	Primers	Primer sequence (5′-3′)	Nucleotide position (5′-3′)	Reference gene	Second T_a_ (°C)
1(PB2)/A	MBTuni-12	ACGCGTGATCAGC**R**AAAGCAGG	1–12	GU907121	59
	PB2_841R24	AGATGCTAGTGGATCTGCTGATAC	864–841		
1(PB2)/B	PB2_778F24	AGGAATGACGATGTTGACCAAAGC	778–801		60
	PB2_1631R24	CAGGACCGTTAATCTCCCACATCA	1654–1631		
1(PB2)/C	PB2_1501F22	GAGAGGGTGGTGGTTAGCATTG	1501–1522		59
	MBTuni-13	ACGCGTGATCAGTAGAAACAAGG	2341–2329		
2(PB1)/A	MBTuni-12	ACGCGTGATCAGC**R**AAAGCAGG	1–12	AB441948	60
	PB1_820R21	CGGAAGTCCAGACTGTTCAAG	840–820		
2(PB1)/B	PB1_733F23	AAARGAAGGGCTATTGCAACACC	733–755		60
	PB1_1765R23	CCTGYCCTTGATTGGGTTTGATC	1787–1765		
2(PB1)/C	PB1_1447F25	ATCAACATGAGCAAAAARAAGTCCT	1447–1471		58
	MBTuni-13	ACGCGTGATCAGTAGAAACAAGG	2341–2329		
3(PA)/A	MBTuni-12	ACGCGTGATCAGC**R**AAAGCAGG	1–12	GU907117	60
	PA_778R25	AAGGTTCAATTTGGGCATTCACTTC	802–778		
3(PA)/B	PA_683F21	CACCGAACTTCTCCTGCCTTG	683–703		58
	PA_1558R24	ATTTACCACGTCTGTGTCATTCCT	1581–1558		
3(PA)/C	PA_1416F23	CATTAACACTGCYCTGCTCAATG	1416–1438		59
	MBTuni-13	ACGCGTGATCAGTAGAAACAAGG	2233–2221		
4(HA)/A	MBTuni-12	ACGCGTGATCAGC**R**AAAGCAGG	1–12	GU907114	61
	HA_1013R22	YCCTGTTGCCAATTTCAGAGTG	1034–1013		
4(HA)/B	HA_873F25	TCAATAATGAGATCAGATGCACCCA	873–897		61
	MBTuni-13	ACGCGTGATCAGTAGAAACAAGG	1762–1750		
5(NP)/A	MBTuni-12	ACGCGTGATCAGC**R**AAAGCAGG	1–12	GU907120	61
	NP_868R18	CGCACAGGCAGGTAGGCA	885–868		
5(NP)/B	NP_753F23	AGCAATGGTGGATCAAGTGAGAG	753–775		60
	MBTuni-13	ACGCGTGATCAGTAGAAACAAGG	1567–1555		
6(NA)/A	MBTuni-12	ACGCGTGATCAGC**R**AAAGCAGG	1–12	GU907119	59
	NA_862R23	ATCTGACACCAGGRTATCGAGGA	884–862		
6(NA)/B	NA_699F25	AGTCRGAATGCGTYTGTATCAATGG	699–723		58
	MBTuni-13	ACGCGTGATCAGTAGAAACAAGG	1466–1454		
7(MP)/A	MBTuni-12	ACGCGTGATCAGC**R**AAAGCAGG	1–12	GU907115	61
	MP_582R23	AGCCATTTGCTCCATAGCCTTAG	604–582		
7(MP)/B	MP_429F21	TGGGGGCTGTAACCACTGAAG	429–449		59
	MBTuni-13	ACGCGTGATCAGTAGAAACAAGG	1027–1015		
8(NS)/A	MBTuni-12	ACGCGTGATCAGC**R**AAAGCAGG	1–12	GU907116	60
	NS_464R22	CTCTTCGGTGAAAGCCCTTAGT	485–464		
8(NS)/B	NS382F21	TGGACCAGGCAATCATGGAGA	382–402		60
	MBTuni-13	ACGCGTGATCAGTAGAAACAAGG	890–878		

The T_aS_ for all the PCR primers ranged between 58 and 61°C. MBTuni-12 and MBTuni-13 primers targeting the 5′ and 3′ ends of each segment were adopted from published methods [21], [27], with nucleotides (in bold) representing the modifications made. Nucleotide R (bold) in the primer sequence indicates a degenerate nucleotide that represents A or G.

### PCR Sensitivity

The 15 RNA samples extracted directly from the clinical samples were of quantification cycle values ranging from 21.0 to 30.56 (equivalent to 2.4×10^3^–1.4×10^6^ viral RNA copies/µL of RNA extract) [24]. All of the gene segments from both the clinical and MDCK-cultured samples collected from 2009–2011 were successfully amplified and appeared as specific and discernible bands on the agarose gel. It was noticed that some gene amplifications additionally produced minor non-specific bands in clinical samples with low viral titers.

### Sequencing

**Figure 1:**
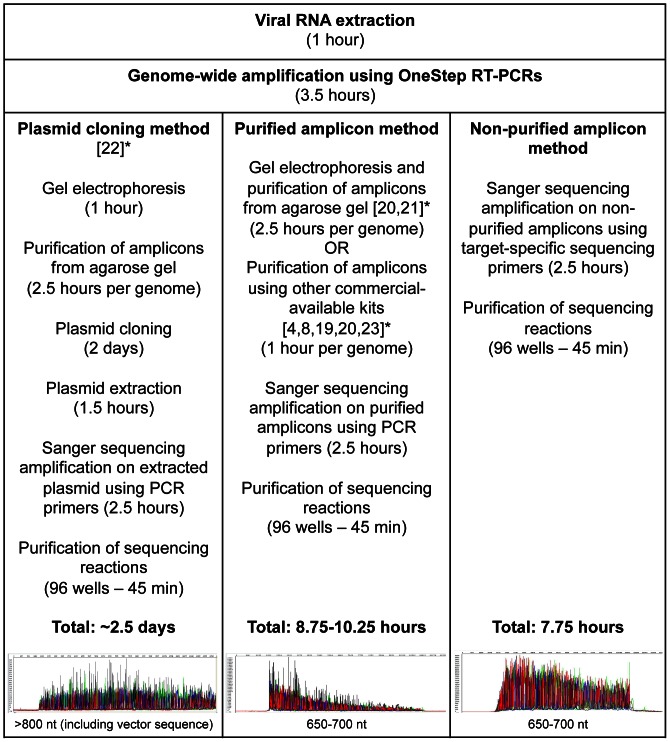
Processing times and steps required for plasmid cloning, purified amplicon, and non-purified amplicon methods. Representative sequencing chromatograms generated from each method are shown. The quality of the raw data obtained from the non-purified amplicon method was comparable with that of the plasmid cloning method. In contrast, the purified amplicon method generated lower quality data in the later portions of the sequence. * Please refer to appropriate references (under the References section).

All the eight segments from the respective 15 clinical and MDCK-cultured samples were successfully sequenced with high Phred quality value (QV) [28], and sequencing length up to 700 bp (Table 1). Length of read (LOR) for all sequence contigs had base calls of QV20 (representing an accuracy of circa one miscall for every 100 bases) and above for at least 20 continuous bases, which was in accordance to the analyzer machine’s default setting. Sequences with a mixture of nucleotides that contained only a single coverage depth was confirmed with reverse sequencing using PCR primers from the purified amplicon method briefly described in Figure 1. In total, the completed sequences obtained from the 15 cultured isolates and directly from the 15 clinical samples covered 408,810 nucleotides, with 13,627 nucleotides per genome, attaining 100% coding completeness. The entire sequencing protocol produced an average of 1.57 sequencing reads covering each nucleotide. Of all the bases in the assembly, an average of 89.49% were QV40 bases (representing an accuracy of circa one miscall for every 10,000 bases) or higher, and an average of 93.46% were QV30 bases (one miscall every 1000 bases) or higher (Table 1). All the sequences were successfully assembled into their respective segments. The use of the non-purified amplicon method resulted in a very high-quality genome assembly, including samples that had Ct values up to 30. The total sequencing raw data obtained per genome was less than 5 megabytes of data storage. The sequence analyses and assembly for each genome was completed within 15–30 minutes. The sequencing chromatograms generated were uploaded into Trace Archive [trace identifier number: 2333373621–2333374798] to allow visual inspection of the traces and quality scores underlying every nucleotide in each of the thirty genomes [29], [30]. All assembled sequences obtained in this study were uploaded onto NCBI GenBank [accession number: JX437693-JX437932].

### Further Testing of Assay Protocol on other Clinical Samples

The genome sequencing and assembling protocols were further tested on 125 additional H3N2 primary clinical samples with Ct values of 30.56 and below. All the 125 samples were collected in NUH as diagnostic samples from 1 May 2009–15 Dec 2012. Of the 125 additional primary clinical samples, 118 were sequenced and assembled completely. In total, 134 out of 140 (96%) primary clinical samples were sequenced successfully in this study with similar Phred quality.

There were seven samples that could not be sequenced completely. More specifically: full PB2, PB1, PA, HA, NP, and NS sequences were not obtainable from 2, 3, 3, 2, 1, 2 of these seven samples, respectively. Of these 13 failures, nine were from two samples with Ct values of 28.72 and 29.04, respectively. The PB1 and PA genes encountered the highest failure rate relative to the others.

## Discussion

Traditionally, Sanger sequencing is performed on purified PCR amplicons to prevent background noise generated during sequencing analyses. Here, it was found possible to employ a non-purified amplicon approach for direct sequencing, which minimized processing time and effort for large-scale viral genome sequencing that produced consistently high quality sequencing data. Figure 1 summarizes the comparisons of the steps and amount of time required to perform sequencing using existing methods (plasmid cloning and purified amplicon approaches) and the non-purified amplicon method employed in this study. Direct sequencing on non-purified amplicons using target-specific sequencing primers not only significantly reduced the workload and cost for the entire genome sequencing, but also produced high quality sequencing peaks that were comparable to those generated by the plasmid cloning method (Figure 1). In addition, it will provide a more economical approach to detect viral mixture or quasispecies because unlike the plasmid cloning method [22], it does not require a minimum critical mass in clone selection for sequencing to obtain representative results. In comparison with the purified amplicon method, this non-purified amplicon method produced much higher quality raw data, according to the data produced from this study (Figure 1).

One possible explanation for the success of this simplified approach may be due to minimum loss of the PCR products as a result of the omission of the purification step, in combination with the use of target-specific sequencing primers that were designed discretely from the PCR primers. Unlike the commonly used M13-flanked PCR primers that allow the use of the M13 primer to sequence the PCR product in a more effective way [4], [31], the independent sequencing primers allowed distinctive sequencing amplification of the specific region of the PCR product, without interference from non-specific products and primer-dimers generated during PCR. To minimize the undesirable effects of residual PCR primers during the sequencing reaction, the forward and reverse primers for each PCR were prepared in equimolar amounts, and PCR conditions of up to 50 total PCR cycles were used, to avoid background noise during sequencing analysis. The 4% (v/v) dimethyl sulfoxide (DMSO) used in the sequencing reaction suppressed background noise encountered by sequencing primer NS373R23 during sequencing analysis [32].

Culturing of clinical samples prior to sequencing is a common practice to obtain sufficient viral genetic material for PCR amplification, as well as to avoid contaminants that may inhibit the PCR. However, it is well-recognized that the passaging of viruses in different hosts may induce excessive host-mediated mutations [33], [34] that can inadvertently lead to biased conclusions. Use of the proposed modified protocol allowed successful complete genome sequencing of human influenza A/H3N2 from clinical and MDCK-cultured samples, from samples with viral loads as low as 2,400 viral RNA copies/µL RNA sample. Assay primer designs based on reference sequences collected from different geographical regions from different periods from 2007–2011, and a 96% success rate of the sequencing of 140 clinical samples collected between 2009–2012 showed that this protocol would be widely applicable to a wide range of viruses. However, further testing on A/H3N2 viruses collected prior to 2009 should be performed to check the sensitivity of this full-genome sequencing assay for these earlier viruses.

The two samples that encountered most failures for individual gene segment sequencing could be possibly due to sample degradation or gene reassortment events within these regions. The H3N2 subtyping results were obtained for the purposes of clinical diagnosis earlier, based on specific real-time RT-PCRs targeting HA and MP genes only. The other five samples that had single incomplete gene sequences may possess single point mutation(s) that affected the capability of the assay to amplify those respective gene targets at either the PCR amplification or sequencing stage.

The entire genomic sequencing for the influenza A/H3N2 virus can be completed with a data storage size of approximately 5 megabytes per genome, permitting convenient data handling by biologists or non-bioinformatics expertise for large-scale sequencing for local surveillance purposes. The sequencing cost per genome of the entire protocol from RNA extraction to sequence analysis was calculated to be less than SGD 350 (∼ USD 290), compared to the conventional purified-amplicon method at around SGD 410 (∼USD 340) and plasmid cloning approach at roughly SGD 1360 (∼USD 1120). The high quality data obtained from multiple sequencing reactions targeting different genes (Table 1) suggested the applicability of this technique for other viral (i.e. small genome) gene sequencing work.

Influenza surveillance will continue on a worldwide basis for the foreseeable future, and molecular surveillance for influenza using partial or full-genome sequencing is now becoming routine in many diagnostic laboratories – especially in those which are not set up to perform the traditional serological surveillance for influenza (hemagglutination inhibition and viral micro-neutralization testing). Among the different seasonal human influenza viruses, influenza A/H3N2 has circulated in the human population since its emergence during the 1968 ‘Hong Kong’ pandemic, and has persisted successfully, despite the emergence of the 2009 A/H1N1pdm virus and its subsequent almost virtual replacement of the previously circulating seasonal influenza A/H1N1 [35], [36]. Ongoing antigenic changes in circulating seasonal A/H3N2 viruses continue to trigger new recommendations for seasonal influenza vaccine composition, to optimize vaccine-induced immunity in both the community and healthcare worker populations [37]–[39]. Thus, ever more efficient and economical methods are required to keep down the costs of molecular surveillance, allowing more laboratories to perform such sequencing routinely, thereby enhancing the quality, temporal and geographical resolution of the local influenza surveillance data available, to keep vaccine manufacturers and public health teams informed [40]. Towards this goal, the simplified sequencing protocol described here has been shown to be effective in obtaining full influenza A/H3N2 genomes at a reasonable price with equipment already available in many diagnostic and research laboratories, suggesting potential use of a similar strategy for studying human influenza A/H1N1pdm viruses.

## Methods

### Ethics Statement

All research studies involving the use of these clinical samples were reviewed and approved by the local institutional ethics review board (National Healthcare Group: B/09/360 and E/09/341).

### Viral RNA Extraction

Viral RNAs were extracted from 200 µL of clinical or cultured samples with either the Qiagen EZ1 Virus mini kit v2.0 or the QIAsymphony Virus/Bacteria mini kit, using their respective proprietary Bio Robot EZ1 and QIAsymphony automated platforms (Qiagen, Valencia, CA), according to the manufacturer’s instructions. All extracted RNAs were eluted into a final volume of 60 µL of elution buffer.

### Reverse Transcription Polymerase Chain Reaction

RT-PCRs were performed with a Superscript III one-step RT-PCR system with Platinum *Taq* high-fidelity polymerase (Invitrogen, Carlsbad, CA). Nineteen RT-PCRs were set up for whole genome amplification. All RT-PCRs were prepared manually in 10 µL of reaction volume, consisting of 5 µL of 2× Reaction Mix, equimolar amounts of forward and reverse primers (0.3 µmol/L each), 0.25 µL of enzyme mix, and 2.5 µL of extracted RNA sample. The remaining volume was topped up with RNase-free water. All RT-PCRs were performed using either the ABI 9700 thermal cycler (Applied Biosystems, CA, USA) or the Biometra T3000 thermocycler (Biometra GmbH, Goettingen, Germany). The cycling conditions were 30 min at 42°C (RT); 2.5 min at 95°C (inactivation of RT enzyme and activation of *Taq* enzyme); 5 cycles of 30 s at 95°C (denaturation), 30 s at 47°C (annealing), and 1.25 min at 68°C (extension); 45 cycles of 30 s at 95°C, 30 s at the respective second annealing temperature (T_a_), and 1.25 min at 68°C; followed by a hold for 10 min at 68°C (final extension). The second T_a_ for each RT-PCR is summarized in Table 2.

### Sequencing

Sequencing reactions were performed directly on non-purified amplicons, using BigDye Terminator v3.1 chemistry (Applied Biosystems). The 10 µL sequencing reaction is composed of 1.5 µL of 5× Buffer, 0.5 µmol/L of respective sequencing primer (Table 1), 1 µL of BigDye enzyme mix, and 1.25 µL of template amplicons. One microliter of 4% DMSO was added into the sequencing reaction together with primer NS373R23 [29]. Large-scale sequencing reactions were carried out on a 96-well plate and purified directly using the BigDyeXTerminator purification kit (Applied Biosystems). Individual sequencing reactions were performed in PCR tubes and purified using the DyeEx 2.0 spin kit (Qiagen). Purified sequencing products were analyzed on the ABI 3130×l genetic analyzer (Applied Biosystems) using the BDx_stdSeq50_POP7_1 run module. Sequencing peak heights were adjusted with the sample injection time ranging from 3–5 seconds.

### Contig Assembly

All sequences were assembled and verified using the ATF software, version 1.0.2.41 (Connexio Genomics, Perth, Australia), using the reference sequence influenza A/Nanjing/1/2009(H3N2) for all segments (GenBank accession: GU907114-GU907117 and GU907119-GU907121), except for the PB1 segment which used influenza A/Sendai-H/F193/2007(H3N2) (GenBank accession: AB441948) as the reference sequence. The primer sequences were subtracted from the data during contig assembly. The multiple A’s observed at the 3′end of the NA, NP, and PA genes were checked carefully by visualization of the sequencing chromatograms.
